# Exploration of Sargassum wightii: Extraction, Phytochemical Analysis, and Antioxidant Potential of Polyphenol

**DOI:** 10.7759/cureus.63706

**Published:** 2024-07-02

**Authors:** Bharathi Selvaraj, Dhanraj Ganapathy

**Affiliations:** 1 Department of Research Analytics, Saveetha Dental College and Hospitals, Saveetha Institute of Medical and Technical Sciences, Saveetha University, Chennai, IND; 2 Department of Prosthodontics, Saveetha Dental College and Hospitals, Saveetha Institute of Medical and Technical Sciences, Saveetha University, Chennai, IND

**Keywords:** ft-ir spectroscopy, antioxidant activity, polyphenols, sargassum wightii, marine environment

## Abstract

Background

The marine environment, with its rich biodiversity and nutrient-dense ecosystems, offers immense potential for discovering novel pharmaceutical products. *Sargassum wightii* is a type of brown seaweed that is particularly abundant in sulfated polysaccharides and polyphenolic compounds. These compounds are renowned for their wide range of biological activities. The exploration of such marine resources is crucial for identifying new compounds that can be harnessed for pharmaceutical and nutraceutical applications.

Aims and objectives

The primary aim of this study is to explore the bioactive compounds present in *S. wightii*, with a specific focus on its polyphenolic content. Additionally, the study seeks to evaluate the antioxidant properties of the compound. By doing so, the research aims to contribute to the growing body of knowledge on marine bioresources and their potential health benefits.

Methods

*S. wightii* samples were collected from the Mandapam coastal region in Rameshwaram, India. The cleaned seaweed was transported to the laboratory, where it was further washed, shade-dried, and ground into a fine powder. The powdered seaweed was then subjected to extraction using four different solvents: n-hexane, dichloromethane, ethyl acetate, and methanol. Phytochemical analyses were conducted on these extracts to identify the presence of various bioactive compounds. The total phenolic content of the extracts was determined, and antioxidant activity was assessed using the phosphomolybdenum method. Functional groups present in the extracts were identified using Fourier Transform Infrared (FT-IR) spectroscopy.

Results

Among the solvents used, the methanol extract yielded the highest amount of crude extract. Phytochemical analysis revealed a variety of bioactive compounds, with the methanol extract showing a notable presence of polyphenols. The total phenolic content was measured at 1.25 ± 0.6 mg gallic acid equivalence (GAE)/g of extract. The antioxidant activity, assessed through the phosphomolybdenum method, demonstrated significant free radical scavenging capabilities with an IC50 (half maximal inhibitory concentration) value of 68.23 ± 3.5 μg/mL. FT-IR spectroscopy confirmed the presence of functional groups characteristic of polyphenols and other bioactive compounds.

Conclusion

The study highlights the significant potential of *S. wightii* as a source of bioactive compounds with substantial antioxidant properties. These findings emphasize the importance of marine algae in the development of pharmaceutical and nutraceutical products, showcasing *S. wightii's* promising role in health-related applications.

## Introduction

The unpredictable nature of the marine environment presents a complex challenge for researchers focused on marine biodiversity and the identification of pharmaceutical products [1]. This environment is highly advantageous due to its extraordinary nutrients and organic materials, which foster remarkable biodiversity, significant physical geography, and increased seafood production beneficial to humans [2]. Key factors in this environment include pH, temperature, salinity, additional nutrients, high organic content, velocity, and abundant carbon and nitrogen sources [3]. Research on predicting marine environmental conditions is limited, with only a few scientists dedicated to this area. Intertidal changes often affect microbes, seaweed, and sponges due to unpredictable environmental factors, which can influence the production of novel biological activities [4]. Compared to terrestrial environments, the marine environment is more complex and nutrient-rich, with organic levels fluctuating unpredictably. These unpredictable conditions can enhance the efficacy of pharmaceutical drugs and affect marine organisms across various ecological levels, from algae to bivalves, crustaceans, and fish. Seaweeds are particularly important among these sources, offering numerous biological properties due to their rich polysaccharides, phytochemical derivatives, and bioactive compounds [5].

Seaweeds are recognized for their abundance of secondary metabolites, including phytochemicals such as tannins, saponins, flavonoids, proteins, steroids, quinones, terpenoids, and cardioglycosides. These compounds hold significant medicinal value and have been extensively used in the pharmaceutical and drug industries. Seaweeds contain vitamins, minerals, and dietary fibers [6]. Due to high nutritional properties, seaweeds can be consumed as basic food in our daily diet but its consumption is limited to coastal parts of the world. Brown seaweed, *Sargassum *spp. is boiled and consumed directly in many seaside countries. It is consumed in India, Japan, and Korea as salad, soups, rice dishes, and savory food ingredients [7]. *Sargassum wightii*, a type of brown algae, is abundant in sulfated polysaccharides known for their diverse biological activities, including free radical scavenging and antioxidant properties. *S. wightii* contains polyphenolic compounds that can help prevent long-term diabetes complications, including cardiovascular diseases, neuropathy, and retinopathy. *S. wightii *(brown) macroalgae have been insufficiently studied concerning the types and properties of their polyphenols. Further research in this area could enhance their exploitation and utilization potential [8]. Polyphenols, a group of natural compounds with phenolic structures, are also found in fruits, vegetables, and other foods and beverages such as tea and chocolate, which are rich sources. Polyphenols from seaweeds have noteworthy potential well-being benefits; they may protect cell constituents against oxidative harm and constrain the chance of degenerative illnesses related to oxidative stretch such as cancer, cardiovascular disease, and osteoporosis [9]. Seaweed polyphenols offer numerous health benefits, including antioxidant and anti-inflammatory effects. They also possess antiviral, antifungal, and antibacterial properties [10]. Additionally, research indicates that these compounds have anti-obesity, anti-hypertensive, anti-diabetic, and anti-cancer activities. Polyphenols from macroalgae are utilized in cosmetic products like skin creams and lotions, where they provide anti-aging, moisturizing, and UV-protective benefits [11]. Polyphenols are recognized for their potent antioxidant properties, capable of counteracting free radicals through the donation of either an electron or a hydrogen atom [12]. Hence, in this study, polyphenols from marine seaweed *S. wightii* were exploited for antioxidant potential.

## Materials and methods

Collection of seaweed

Brown seaweed (*S. wightii*) was gathered from the Mandapam coastal region in Rameshwaram, India during the spring season (in March 2023). The samples were meticulously cleaned with seawater to remove extraneous materials such as epiphytes, sand, pebbles, and shells. Subsequently, the seaweed was promptly transported to the laboratory in a sterile bag. There, the samples were thoroughly washed with tap water followed by distilled water, and then shade-dried at room temperature. The dried seaweed was cut into small pieces and ground into a fine powder using a mixer. The powdered samples were stored in airtight containers for further analysis [13].

Extraction of phytoconstituents using different solvents

The polyphenol was extracted by adding 25 g of dried seaweed in 100 mL of solvents, viz., n-hexane, dichloromethane (DCM), ethyl acetate, and methanol respectively. The mixture was kept overnight in a shaking incubator. The mixture was filtered through Whatman filter paper no.4 and filtrate was allowed to evaporate. The extracts of respective solvents were used for further analysis [14]. The yield of 25 g powder was calculated by using the formula:

Yield = Amount of product/Amount of sample ×100

Phytochemical analysis of the extract

The phytochemical analysis of *S. wightii *extracts was performed to reveal the presence or absence of several constituents, including alkaloids, anthocyanins, combined anthraquinones, cardiac glycosides, coumarins, flavonoids, glycosides, phenols, phylobatannins, quinones, reducing sugars, saponins, steroids, tannins, and terpenoids [15,16]. 

Estimation of total phenolic content

A 100 µl aliquot of the crude sample was mixed with 2 mL of 2% sodium carbonate and left to stand in the dark at room temperature for 2 min. A standard solution of 30 mg/mL gallic acid was used to prepare a calibration curve with concentrations ranging from 10-20 mg/l. The phenolic content was expressed as gallic acid equivalence (GAE)/g of extract. The absorbance of all sample solutions was measured at 720 nm using a spectrophotometer [17].

Total antioxidant capacity by the phosphomolybdenum method

The scavenging activity of the seaweed extract was assessed using the phosphomolybdenum method. A mixture of 2 mL of the extract and 1 mL of reagent solution containing 4 mM ammonium molybdate, 28 mM sodium phosphate, and 0.6 M sulfuric acid was prepared. The reaction solution was then capped and incubated in a water bath at 95°C for 90 minutes. After cooling the samples to room temperature, the antioxidant activity was quantified as the GAE/g of dried extract. The absorbance of the solution was measured at 635 nm using a spectrophotometer against a blank. The percentage inhibition was calculated using a formula, and IC50 (half maximal inhibitory concentration) values were determined using GraphPad Prism software (GraphPad Software, Inc., San Diego, CA) [18].

Characterization of seaweed extract

*Fourier Transform Infrared (FT-IR) Spectroscopy* *Analysis*

FT-IR analysis was conducted to identify potential biomolecules present in the polyphenol extraction from marine brown algae *S. wightii*. Twenty milligrams of the crude sample were mixed with four different filtered solvent extractions (n-hexane, dichloromethane, ethyl acetate, and methanol) and analyzed using FT-IR spectroscopy. The analysis was performed at the Department of Sophisticated Analytical Instrument Facility (SAIF), IIT campus, Chennai. FT-IR spectra were recorded within the range of 400-4,000 cm-1. Various stretching vibration modes were identified and assigned to determine the functional groups present in the polyphenol extraction from marine brown algae.

## Results

Collection and extraction of seaweed *S. wightii*

The collected brown algae *S. wightii *with a distinctive macroscopic appearance characterized by its bushy, branched structure. The branches bear small, leaf-like structures called blades, which are elongated and often have a slightly serrated edge. Air bladders, or vesicles, are found along the branches, aiding in buoyancy. The overall color ranges from dark brown to olive green. Table 1 illustrates the extraction yields from *S. wightii* using various solvents, showing that different solvents produced varying amounts of crude extract. The methanol extract yielded the highest amount whereas dichloromethane had the lowest yield.

**Table 1:** Extraction yields of S. wightii extracted using different solvents Data (n=3) are presented as the mean ± SEM

Solvent	Average % yield
n-Hexane	25.2±0.7
Dichloromethane	11.7±0.2
Ethyl acetate	27.2±0.6
Methanol	37.3±0.3

Phytochemical analysis of the extract

Saponins were found to be extracted by all four solvents. Steroids were extracted with all the solvents except methanol. Cardioglycosides were present in all the extracts except those obtained with n-hexane. The methanol extract and ethylacetate showed the presence of four compounds each whereas n-hexane and dichloromethane showed the presence of three compounds each (Table 2).

**Table 2:** Phytochemical analysis of various solvent extracts of S. wightii “+” – indicates presence; “-” – indicates absence

S.No	Name of the compound	n-hexane	Dichloromethane (DCM)	Ethyl acetate	Methanol
1.	Tannins	_	_	+	_
2.	Saponins	+	+	+	+
3.	Flavonoids	_	_	_	_
5.	Steroids	+	+	+	_
6.	Poly phenols	_	_	_	+
7.	Quinones	_	_	_	_
8.	Terpenoids	+	_	_	+
9.	Cardioglycosides	_	+	+	+

Total polyphenolic content

In this study, the total phenolic content of the marine seaweed *S. wightii* was found to be 1.25 ± 0.6 mg GAE/g of dried extract. This measurement indicates the concentration of phenolic compounds, which are known for their antioxidant properties. The value of 1.25 mg GAE/g suggests a significant presence of these compounds, which contribute to the seaweed's potential health benefits, including anti-inflammatory, antimicrobial, and anticancer activities. This result underscores the potential of *S. wightii *as a source of valuable phenolic compounds for pharmaceutical and nutraceutical applications.

Antioxidant activity

Antioxidants neutralize free radicals such as hydroxyl and peroxyl ions, preventing these reactive species from causing oxidative damage. In this study, the antioxidant capacity of the *S. wightii *extract was evaluated and compared to that of gallic acid, a known antioxidant. The IC50 value, which indicates the concentration required to inhibit 50% of the free radical activity, was determined to be 68.23 ± 3.5 μg/mL for the seaweed extract and 73.81 ± 6.3 μg/mL for gallic acid. These IC50 values reflect the potency of the antioxidants, with lower values indicating higher effectiveness.

The results reveal that *S. wightii *extract has a slightly higher antioxidant activity than gallic acid, as evidenced by its lower IC50 value. The reducing power, which measures the ability of the antioxidants to donate electrons and neutralize free radicals, was also assessed. The findings showed that the reducing power followed the order: gallic acid > *S. wightii *extract. This indicates that while both substances are effective antioxidants, gallic acid exhibits a marginally higher reducing power compared to the seaweed extract.

FT-IR spectroscopic analysis

**Figure 1:**
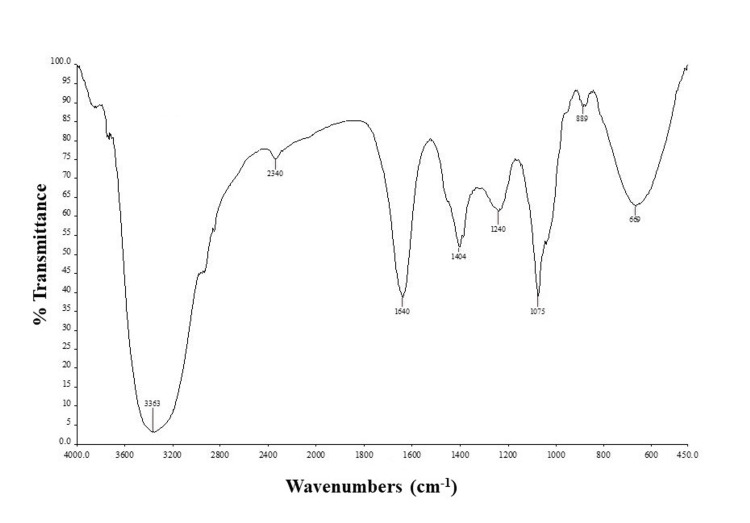
FT-IR analysis of polyphenol extracted from marine brown seaweed S. wightii FT-IR - Fourier transform infrared

FT-IR analysis was conducted on the polyphenol extraction from marine brown algae *S. wightii* to determine the functional groups present (Figure 1). The FT-IR spectrum provided a detailed fingerprint of the molecular components, revealing several characteristic peaks. A broad band observed at 3,363 cm^-1 ^was attributed to O-H stretching vibrations, indicative of hydrogen-bonded alcohol and phenol groups. This peak is significant as it confirms the presence of phenolic compounds, which are known for their antioxidant properties. The peak at 1,640 cm^-1^ corresponded to C=C stretching vibrations typical of alkenes. This peak suggests the presence of unsaturated compounds, which are often involved in various biological activities. A peak at 1,404 cm^-1^ was assigned to C-H stretching vibrations of alkanes, which indicated the presence of simple hydrocarbon chains within the extract. Additional peaks at 1,240 cm^-1^ and 1,075 cm^-1^ were associated with C-H deformation and C-O or C-C stretching vibrations, respectively. These peaks are characteristic of carbohydrates and polysaccharides, confirming the presence of these compounds in the extract. The spectrum also showed a peak at 889 cm^-1^, which can be attributed to C-H bending of glucose or galactose units, and a peak at 669 cm^-1^, which is related to C-S stretching vibrations of sulfates. These results highlight the complex composition of the *S. wightii* extract, containing a rich array of phenolic compounds, carbohydrates, and polysaccharides. The presence of these functional groups underscores the potential of *S. wightii* for various biological applications, particularly due to its antioxidant properties conferred by phenolic compounds and its potential health benefits related to carbohydrates and polysaccharides.

## Discussion

Plant substances continue to be a vital source of medications for the global population, with numerous plant-based drugs currently in widespread clinical use. Over the past few decades, many plants have been commonly utilized for various treatments. In recent years, however, the significance of marine organisms as a source of novel substances has been increasingly recognized. The unique metabolic and physiological adaptations of aquatic organisms, which enable them to thrive in diverse and complex habitats, offer significant potential for producing secondary metabolites absent in terrestrial environments. Consequently, marine algae are one of the richest sources of known and novel bioactive compounds [19]. Hence, this study focuses on the utilization of marine algae *S. wightii* for their bioactive metabolites in particular polyphenols for their antioxidant potential.

The nutraceutical potentials of macroalgae vary based on species, season, temperature, climate, water salinity, and sunlight exposure. Additionally, the concentration and polarity of the solvent used in extracting phytochemical compounds significantly influence the outcomes [20]. The extraction yields of *S. wightii* using various solvents were performed and the methanol extract yielded the highest amount (Table 1). The study demonstrated that all four solvents extracted saponins, while steroids were present except methanol, and cardio glycosides were absent in the n-hexane extract. Overall, seaweeds, including *S. wightii*, are rich sources of biologically active compounds, making them valuable for various pharmaceutical and nutraceutical applications (Table 2) [14]. Among the phytoconstituents, polyphenols are compounds characterized by one or more aromatic rings with hydroxyl groups. They have demonstrated antioxidant, antimicrobial, antidiabetic, anti-inflammatory, and anticancer properties in both vitro and in vivo studies [21]. The health benefits of phenolic compounds are well-recognized in the prevention of cardiovascular diseases, cancers, and neurological disorders [22]. Due to the therapeutic potential of marine polyphenols, there is increasing interest in creating nutraceuticals and pharmaceuticals that incorporate these compounds as active ingredients [11]. In this, the total phenolic compound of marine seaweed *S. wightii* was found to be 1.25±0.6 mg GAE/g. Kumar et al. [8] compared the total polyphenols present in the marine seaweeds *S. wightii* and *U. rigida *and found that *S. wightii *has more polyphenols. Kumar et al. [23] proposed that in *S. wightii*, a polyphenol called phlorotannin in a methanol extract underwent structural changes during polymerization. This process led to the formation of various subunits, including fucols, fucophlorethols, eckol, and fuhalols, thereby increasing the phenolic content. 

Antioxidants quench the free radicals, reducing the aging process and cell damage. Antioxidant plays a key role in cancer prevention and rapid cell proliferation. Seaweeds are used as the best food source since it has rich antioxidants that inhibit the reactive oxygen species. Seaweeds have much less fat content but are rich in minerals and vitamins. They produce bioactive secondary metabolites like alkaloids, terpenoids, and polysaccharides which are highly biomedical significant to treat many ailments [24]. Antioxidants quench the free radicals like hydroxyl, and peroxyl ions thereby preventing the free radical ions from getting oxidized. The phosphomolybdenum method offers a straightforward and cost-effective alternative for evaluating total antioxidant capacity, utilizing inexpensive reagents compared to existing methods [25]. The IC50 value of the seaweed extract and gallic acid was found to be 68.23 ±3.5 μg/mL and 73.81± 6.3 μg/mL, respectively. The reducing power of the samples was shown and it was found to be in the following order gallic acid, *S. wightii* extract. Khan et al. [18] revealed the antioxidant property of *Launaea procumbens* extract and found the value of IC50 64.27 ± 2.1 μg/mL and ascorbic acid with IC50 72.3 ± 2.2 μg/mL. The difference in the antioxidant activity of different plant extracts might be the difference in the phytoconstituents present in the extract. FT-IR analysis was conducted on the polyphenol extraction from marine brown algae *S. wightii* to determine the functional groups present (Figure 1). The FT-IR spectrum revealed several peaks, including a broad band at 3,363 cm^-1 ^corresponding alcohol and phenol, a peak at 1,640 cm^-1^ indicating C=C stretching vibration, and another peak at 1,404 cm^-1 ^signifying alkanes, which suggested the presence of phenolic groups [8]. The peak observed in 1,240 cm^-1^ and 1,075 cm^-1^ can be attributed to carbohydrates and polysaccharides. The peak at 889 cm^-1^ contributed to glucose or galactose and the peak at 669 cm^-1^ was attributed to the C-S stretching of sulfates [26]. The valued traditional pharmacological properties of Mentha herbs stem from their aromatic nature and are associated with the presence of bioactive phytochemicals like phenolic compounds, tannins, terpenes, terpenoids, quinones, coumarins, flavonoids, alkaloids, sterols, and saponins [27]. Chilli peppers exhibit antioxidant activity due to the presence of various compounds, including vitamins C and E, anthocyanins, and notably, phenolic compounds. These phenolic compounds function as hydrogen atom donors, effectively neutralizing and scavenging free radicals within the body [28]. Similar to this, our study also reveals the presence of bioactive molecules, viz., saponins, polyphenols, terpenoids, and cardio glycosides by phytochemical and FT-IR analysis. The data from the current study indicate that *S. wightii* contains significant amounts of phenolic compounds with potent antioxidant properties. Numerous studies have documented those terrestrial plants, which possess free radical scavenging and antioxidant properties, are employed in treating diseases such as cancer, tissue inflammation, and cardiovascular disease [29]. Additionally, there has been a surge in publications highlighting the health benefits of polyphenols from different sources with enhanced antioxidant potential. The free radical scavenging methods used in this study were straightforward and yielded reproducible results, demonstrating the antioxidant properties of marine seaweed *S. wightii*. It is concluded that the observed antioxidant capacity of *S. wightii* is likely due to the high presence of phenolic compounds with possible pharmacological applications in treating renal injuries, hormonal and sexual disorders, and antimicrobial applications.

This manuscript sheds light on the substantial potential of marine seaweed *S. wightii*, highlighting its abundance in bioactive compounds, notably polyphenols, renowned for their powerful antioxidant properties. Despite the promising findings, the study faces certain limitations and challenges. The variability in the chemical composition of *S. wightii* due to seasonal and environmental factors may affect the consistency of bioactive compound yields. The methanol extract showed notable antioxidant activity, the in vitro results may not fully translate to in vivo efficacy due to differences in bioavailability and metabolism. Furthermore, purification and structural characterization techniques are needed for a deeper understanding of the specific compounds responsible for the observed bioactivities. These challenges highlight the need for more comprehensive and controlled studies to fully realize the pharmaceutical potential of *S. wightii*.

## Conclusions

This study successfully demonstrated the potential of the brown seaweed *S. wightii* as a rich source of bioactive compounds, particularly polyphenols, with significant antioxidant properties. The extraction using methanol yielded the highest amount of crude extract, and the phytochemical analysis confirmed the presence of various beneficial compounds. The total phenolic content was substantial, and the antioxidant activity showed effective free radical scavenging. FT-IR analysis further identified key functional groups, supporting the presence of valuable phytochemicals. These results suggest that *S. wightii *holds promise for developing nutraceutical products, emphasizing the importance of marine resources for possible application in the discovery of novel bioactive compounds.
